# Enriched environment ameliorates fear memory impairments induced by sleep deprivation via inhibiting PIEZO1/calpain/autophagy signaling pathway in the basal forebrain

**DOI:** 10.1111/cns.14365

**Published:** 2023-07-23

**Authors:** Zi‐qing Zhang, Yan Lu, Hao Zhang, Su‐he Dong, Ya‐tong Wu, Si‐nian Wang, Ai‐hua Huang, Qi‐sheng Jiang, Shi‐min Yin

**Affiliations:** ^1^ The Postgraduate Training Base of Jinzhou Medical University (The PLA Rocket Force Characteristic Medical Center) Beijing China; ^2^ Department of Neurology The PLA Rocket Force Characteristic Medical Center Beijing China; ^3^ Department of Anesthesiology The PLA Rocket Force Characteristic Medical Center Beijing China; ^4^ Department of Nuclear Radiation Injury and Monitoring The PLA Rocket Force Characteristic Medical Center Beijing China

**Keywords:** autophagy, basal forebrain, fear memory, PIEZO1, sleep deprivation

## Abstract

**Aims:**

To verify the hypothesis that an enriched environment (EE) alleviates sleep deprivation‐induced fear memory impairment by modulating the basal forebrain (BF) PIEZO1/calpain/autophagy pathway.

**Methods:**

Eight‐week‐old male mice were housed in a closed, isolated environment (CE) or an EE, before 6‐h total sleep deprivation. Changes in fear memory after sleep deprivation were observed using an inhibitory avoidance test. Alterations in BF PIEZO1/calpain/autophagy signaling were detected. The PIEZO1 agonist Yoda1 or inhibitor GsMTx4, the calpain inhibitor PD151746, and the autophagy inducer rapamycin or inhibitor 3‐MA were injected into the bilateral BF to investigate the pathways involved in the memory‐maintaining role of EE in sleep‐deprived mice.

**Results:**

Mice housed in EE performed better than CE mice in short‐ and long‐term fear memory tests after sleep deprivation. Sleep deprivation resulted in increased PIEZO1 expression, full‐length tropomyosin receptor kinase B (TrkB‐FL) degradation, and autophagy, as reflected by increased LC3 II/I ratio, enhanced p62 degradation, increased TFEB expression and nuclear translocation, and decreased TFEB phosphorylation. These molecular changes were partially reversed by EE treatment. Microinjection of Yoda1 or rapamycin into the bilateral basal forebrain induced excessive autophagy and eliminated the cognition‐protective effects of EE. Bilateral basal forebrain microinjection of GsMTx4, PD151746, or 3‐MA mimicked the cognitive protective and autophagy inhibitory effects of EE in sleep‐deprived mice.

**Conclusions:**

EE combats sleep deprivation‐induced fear memory impairments by inhibiting the BF PIEZO1/calpain/autophagy pathway.

## Graphical abstract

Fear memory acquisition and maintenance are regulated by basal forebrain PIEZO1/calpain signaling, which functions through regulating BDNF/TrkB signaling and autophagy. Sleep deprivation increases PIEZO1/calpain signaling activity in the basal forebrain and induces short‐ and long‐term fear memory impairments. Enriched environments (EE) rescues sleep deprivation‐induced fear memory impairments by inhibition of PIEZO1/calpain/autophagy signaling.

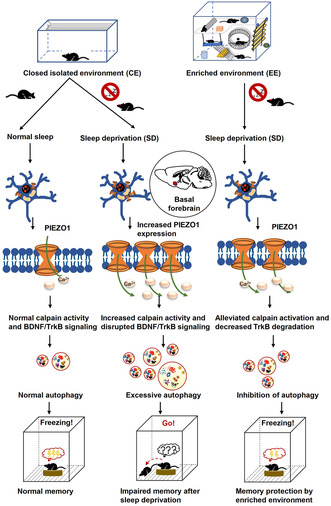

## 1 INTRODUCTION

Sleep deprivation is the subjective feeling of sleep deficiency. In the United States, approximately one‐third of the population is at risk of sleep deficiency.^1^ The National Health and Nutrition Examination Survey (2017–2020) among adults aged 20 years or older in the United States showed that the mean sleep debt was 0.73 h for all people and 30.5% of adults experienced 1 h or more of sleep debt, and the prevalence of daytime sleepiness was 27.2%.^2^ Moreover, the prevalence of sleep deficiency due to short sleep duration is increasing irrespective of ethnic background.^3^, ^4^ Sleep deprivation damages the central nervous system and causes or aggravates cognitive impairment.^5^, ^6^, ^7^, ^8^ The external environment regulates both sleep and cognition. Social separation or isolation significantly increases the incidence of disturbed sleep^9^ and cognitive impairment.^10^ A closed isolated environment (CE) significantly enhances cognitive impairments caused by sleep deprivation, especially attention and complex task performance.^11^ Conversely, positive incentive stimuli counteracted the sleep deprivation‐induced decrease in emotional working memory.^12^ Animal studies have also found that enriched environment (EE) dramatically reduces cognitive impairment after sleep deprivation.^13^ These studies suggest that EE may help reduce the cognitive decline attributed to acute sleep deprivation, but the specific brain areas involved and the associated signaling mechanisms remain unclear.

The basal forebrain, enriched in cholinergic neurons, is one of the critical brain regions for sleep and learning memory regulation.^14^, ^15^ Compared to standard care, EE reduced cholinergic neuron loss in the basal forebrain medial septal area due to traumatic brain injury (TBI) and promoted spatial learning and memory capacity.^16^ In mice with poststroke cognitive impairment, EE improved cognitive performance and enhanced the basal forebrain‐hippocampal cholinergic neural circuits.^17^ These studies suggest that the basal forebrain may be the brain region where EE acts on. However, it remains unclear whether the basal forebrain mediates the cognitive protective effects of EE under sleep deprivation.

PIEZO1 is a mechano‐gated ion channel that mediates signal transduction mainly by permeabilizing positive ions, such as calcium (Ca^2+^).^18^ Our previous study demonstrated that acute sleep deprivation increases the enzymatic cleavage of TrkB‐FL, a receptor for brain‐derived neurotrophic factor (BDNF), through activation of the basal forebrain PIEZO1/Ca^2+^/calpain pathway, leading to curtailed BDNF signaling and memory impairments.^19^, ^20^ It is unclear whether EE exerts cognitive protective effects by modulating this signaling pathway.

Neuronal autophagy is involved in memory consolidation.^21^, ^22^ Furthermore, impaired neuronal autophagy in the basal forebrain may also be associated with the pathogenesis of Alzheimer's disease,^23^ and BDNF inhibits neuronal autophagy, regulates synaptic plasticity, and induces memory consolidation by activating TrkB/PI3K/Akt signaling.^24^ Sleep deprivation is accompanied by excessive autophagy of hippocampal neurons, which participate in sleep deprivation‐induced memory impairments.^21^, ^25^ There have been no reports of autophagy in basal forebrain neurons involved in sleep deprivation‐induced memory deficits. Considering that PIEZO1 mainly acts through Ca^2+^ signaling,^18^ which can regulate autophagy through calpain, calcineurin, and CaMKKβ,^26^, ^27^ we proposed and tested the hypothesis that EE may regulate PIEZO1/calpain/autophagy signaling pathway in the basal forebrain of mice to alleviate sleep deprivation‐induced memory impairments.

## 2 MATERIALS AND METHODS

### 2.1 Animals

The animals used were 8‐week‐old C57BL/6J male mice, weighing approximately 20–25 g purchased from SPF Biotechnology Co. Mice were housed with a fixed 12‐h light/12‐h dark diurnal cycle, at a controlled ambient temperature of 23°C ± 3°C, with ad libitum access to food and water. All procedures were performed according to the National Institutes of Health Guide for the Care and Use of Laboratory Animals. The Ethics Committee of the PLA Rocket Force Characteristic Medical Center approved the experimental procedure (KY2021101).

A total of 268 mice were used in this experiment. Among them, 12 mice were excluded due to training failure in the step‐down inhibitory avoidance test, 20 were excluded due to catheter dislodgement before drug administration, and 58 were excluded due to the wrong basal forebrain microinjection sites. Finally, data from 178 mice were analyzed.

### 2.2 Experimental design

**FIGURE 1:**
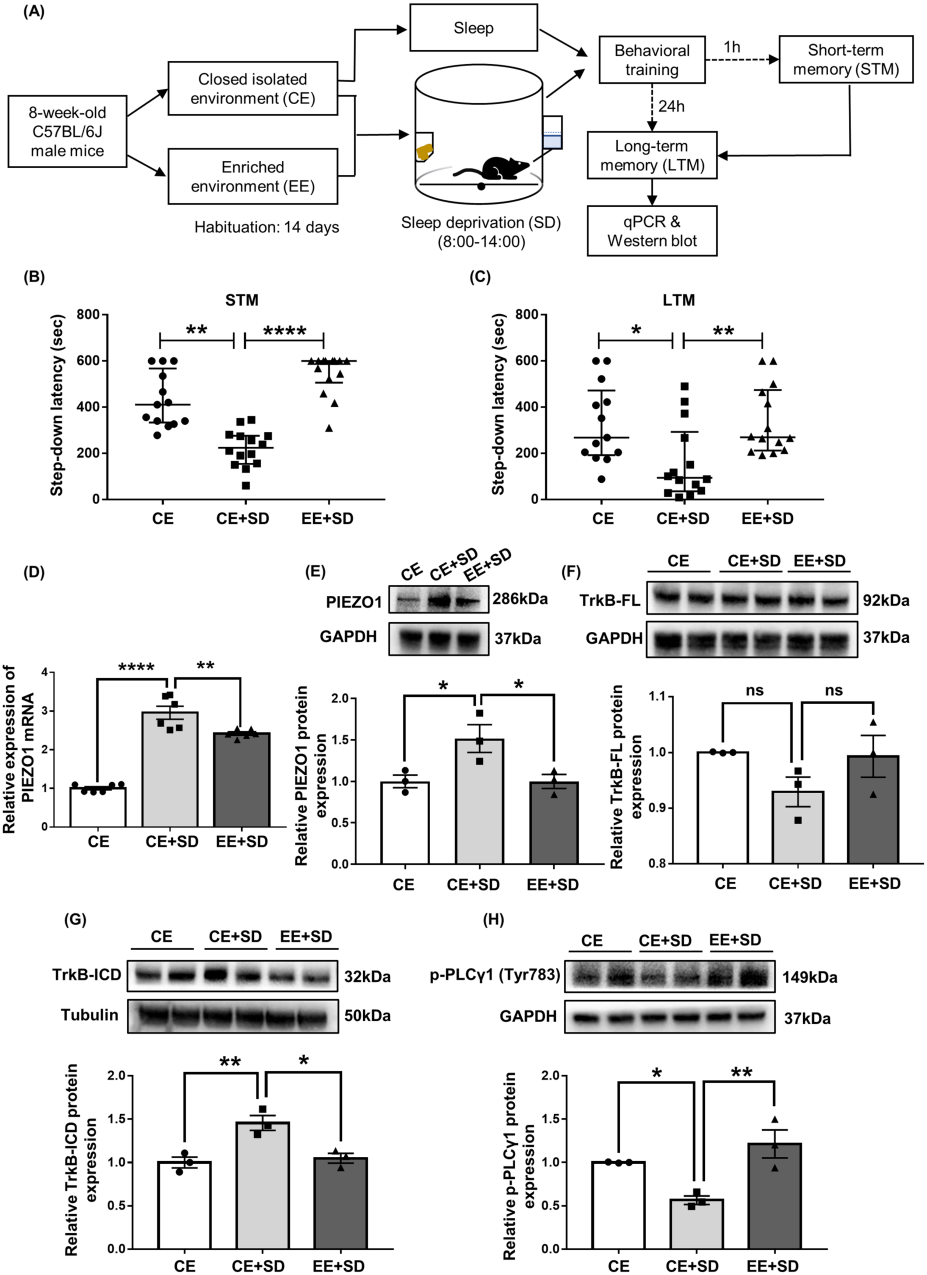
Enriched environment counteracts sleep deprivation‐induced fear memory impairments and enhancement of PIEZO1 signaling. (A) Experimental design. Animals underwent 6 h of total sleep deprivation (SD) after 14 days of enriched environment (EE) or closed isolated environment (CE) housing. Short‐term (1 h after inhibitory avoidance training) and long‐term (24 h after training) fear memory performance was assessed following sleep deprivation. Changes in PIEZO1 expression and BDNF/TrkB/ PLCγ1 signaling were examined. (B, C) EE reverses short‐term (B) and long‐term (C) fear memory impairments induced by acute sleep deprivation. (D, E) EE reverses the increase in PIEZO1 mRNA and protein expression induced by acute sleep deprivation. (F) EE and sleep deprivation do not affect full‐length TrkB expression. (G) EE reverses the degradation of TrkB into TrkB‐ICD induced by acute sleep deprivation. (H) EE reverses the inhibition of PLCγ1 phosphorylation caused by acute sleep deprivation. Thirteen mice in the CE group and 14 mice in each of the CE + SD and EE + SD groups were used in behavioral experiments. PCR and Western blot experiments were performed in three independent samples. Values were expressed as medians (interquartile ranges) or means ± SEMs. *p < 0.05; **p < 0.01; ***p < 0.001; ****p < 0.0001.

**FIGURE 2:**
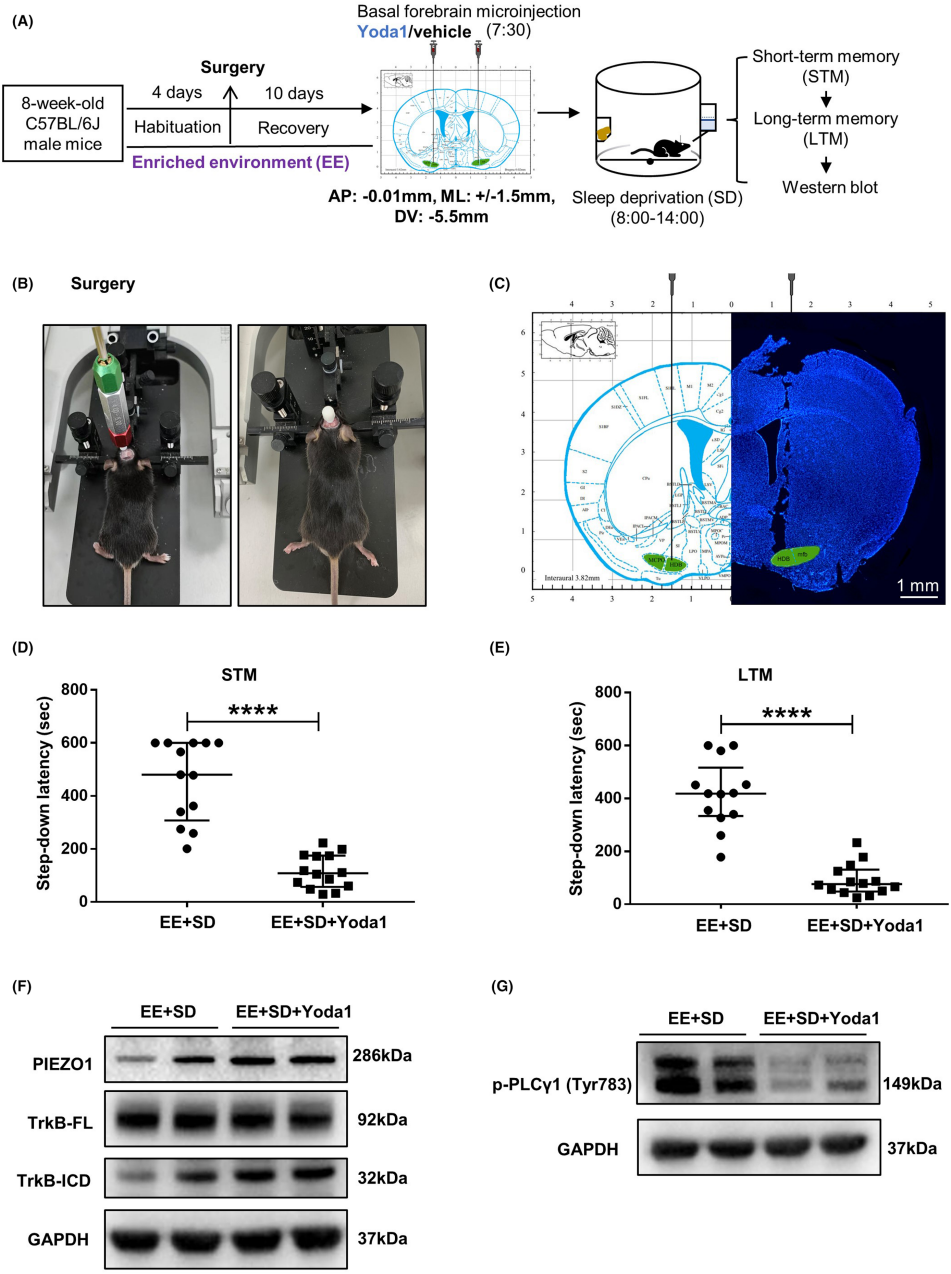
PIEZO1 activator eliminates the ameliorative effect of EE on fear memory impairments due to sleep deprivation. (A) Experimental design. The PIEZO1 activator Yoda1 (250 ng/250 nL/side) or vehicle was bilaterally injected into the basal forebrain before sleep deprivation. (B) Stereotactic surgery diagram. Bilateral guide tubes were preplaced over the basal forebrain using stereotactic surgery. (C) Representative image of the microinjection probe tip location, depicting the targeted basal forebrain region (green). The right side shows a DAPI‐stained image of the needle tract trajectory, consistent with the schematic diagram of a coronal brain slice from The Mouse Brain in Stereotaxic Coordinates‐3rd edition. (D, E) Yoda1 induces short‐term (D) and long‐term (E) fear memory impairments following sleep deprivation in EE mice. (F) Yoda1 increases PIEZO1 protein expression in sleep‐deprived EE mice. Yoda1 has no effect on full‐length (TrkB‐FL) expression (F), but increases TrkB degradation into TrkB‐ICD (F), and inhibits PLCγ1 activation (G). Thirteen mice in the EE + SD group and 14 mice in the EE + SD + Yoda1 group were used. Western blot experiments were performed in three independent samples. Values are expressed as medians (interquartile ranges) or means ± SEMs. *p < 0.05; **p < 0.01; ****p < 0.0001.

**FIGURE 3:**
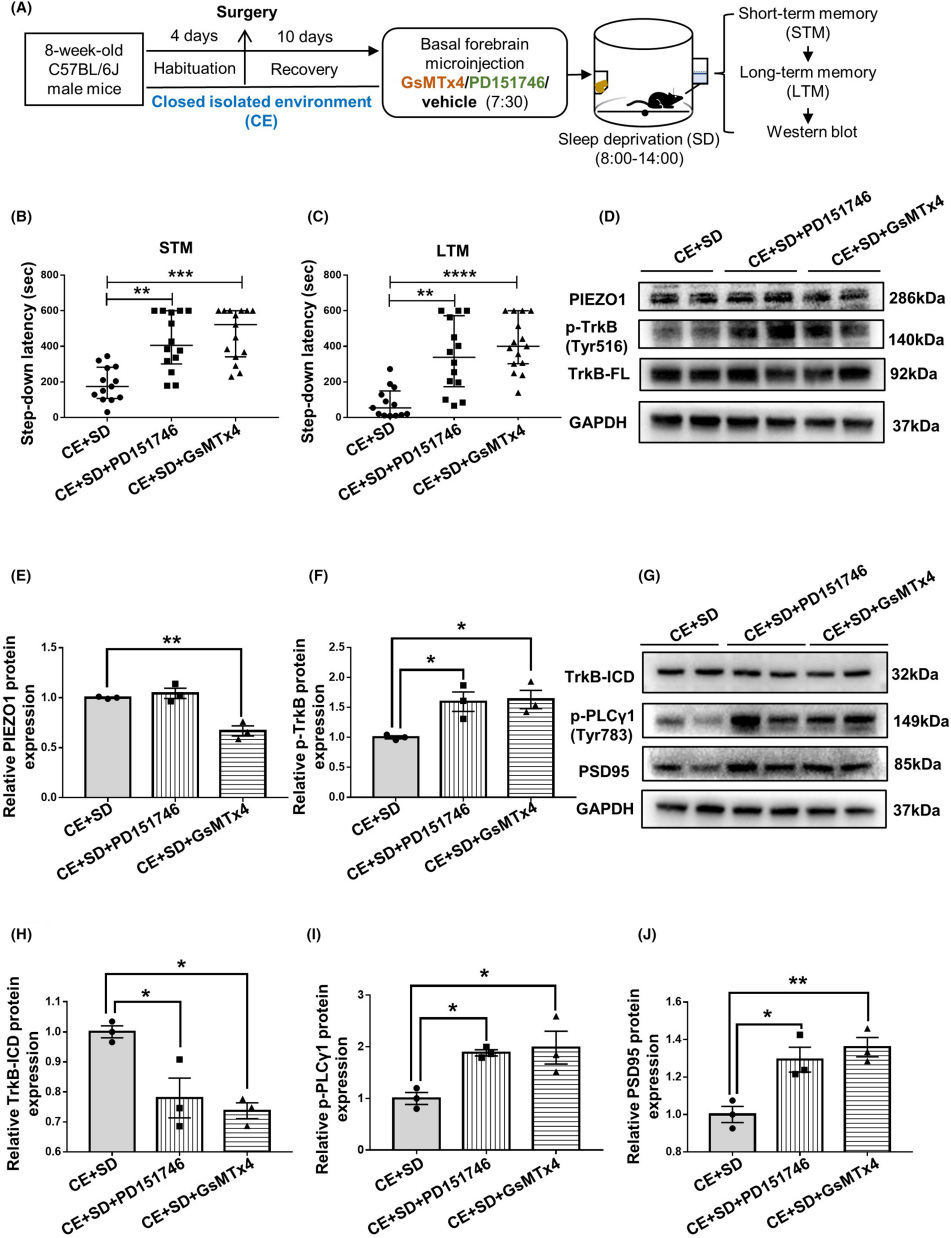
Inhibition of PIEZO1 or calpain inhibitor mimics the ameliorative effect of EE on sleep deprivation‐induced fear memory impairments. (A) Experimental design. The PIEZO1 inhibitor GsMTx4 (300 ng/250 nL/side) or calpain inhibitor PD151746 (2 μg/250 nL/side) or vehicle was microinjected into the bilateral basal forebrain of CE mice. (B and C) GsMTx4 or PD151746 counteracts sleep deprivation‐induced short‐term (B) and long‐term (C) fear memory impairments. (D–J) Changes in PIEZO1, TrkB‐ICD, PSD95 protein translation, and TrkB and PLCγ1 phosphorylation levels were detected by Western blot. Statistical histograms were plotted. Thirteen mice in the CE + SD group, 14 mice in the CE + SD + PD151746 group, and 15 mice in the CE + SD + GsMTx4 group were used. Western blot experiments were performed in three independent samples. Values are expressed as medians (interquartile ranges) or means ± SEMs. *p < 0.05; **p < 0.01; ****p < 0.0001.

**FIGURE 4:**
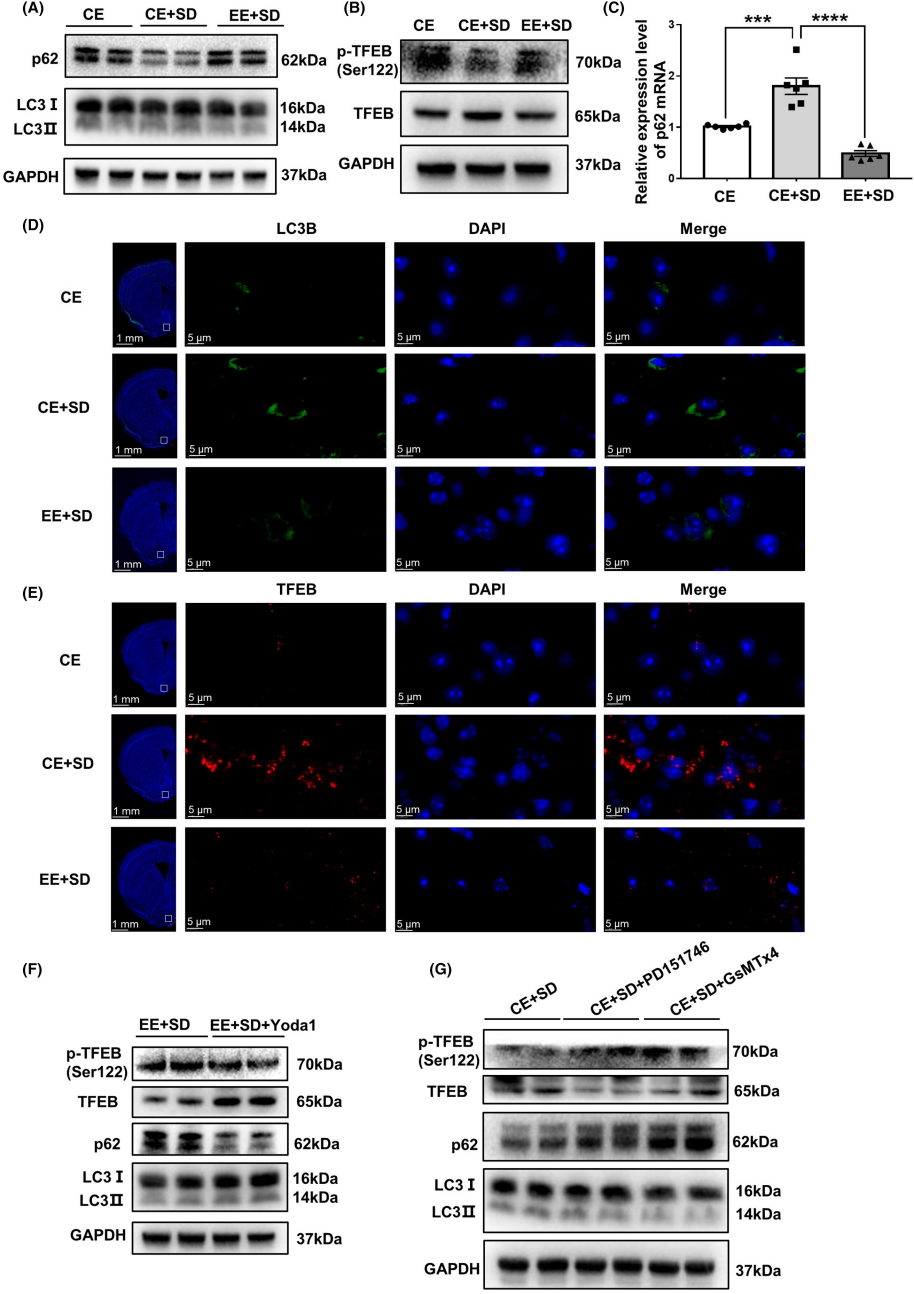
EE or inhibition of PIEZO1/calpain signaling alleviates excessive autophagy caused by sleep deprivation. (A, B) EE reverses excessive autophagy caused by sleep deprivation, as evidenced by the increased LC3 II/I ratio and enhanced p62 degradation (A), increased TFEB protein levels, and reduced TFEB phosphorylation levels (B). (C) EE alleviates p62 mRNA overexpression. (D, E) EE alleviates the sleep deprivation‐induced enhancement of LC3B (D) and TFEB (E) fluorescence intensity in the basal forebrain. (F) Yoda1 induces increased LC3 activation, decreased p62, increased TFEB, and decreased TFEB phosphorylation. (G) Inhibition of the PIEZO1/calpain signaling pathway in the basal forebrain of sleep‐deprived CE mice leads to decreased LC3 activation, increased p62, decreased TFEB, and increased TFEB phosphorylation. *n* = 3 per group. Values are expressed as means ± SEMs. *p < 0.05; **p < 0.01; ***p < 0.001.

**FIGURE 5:**
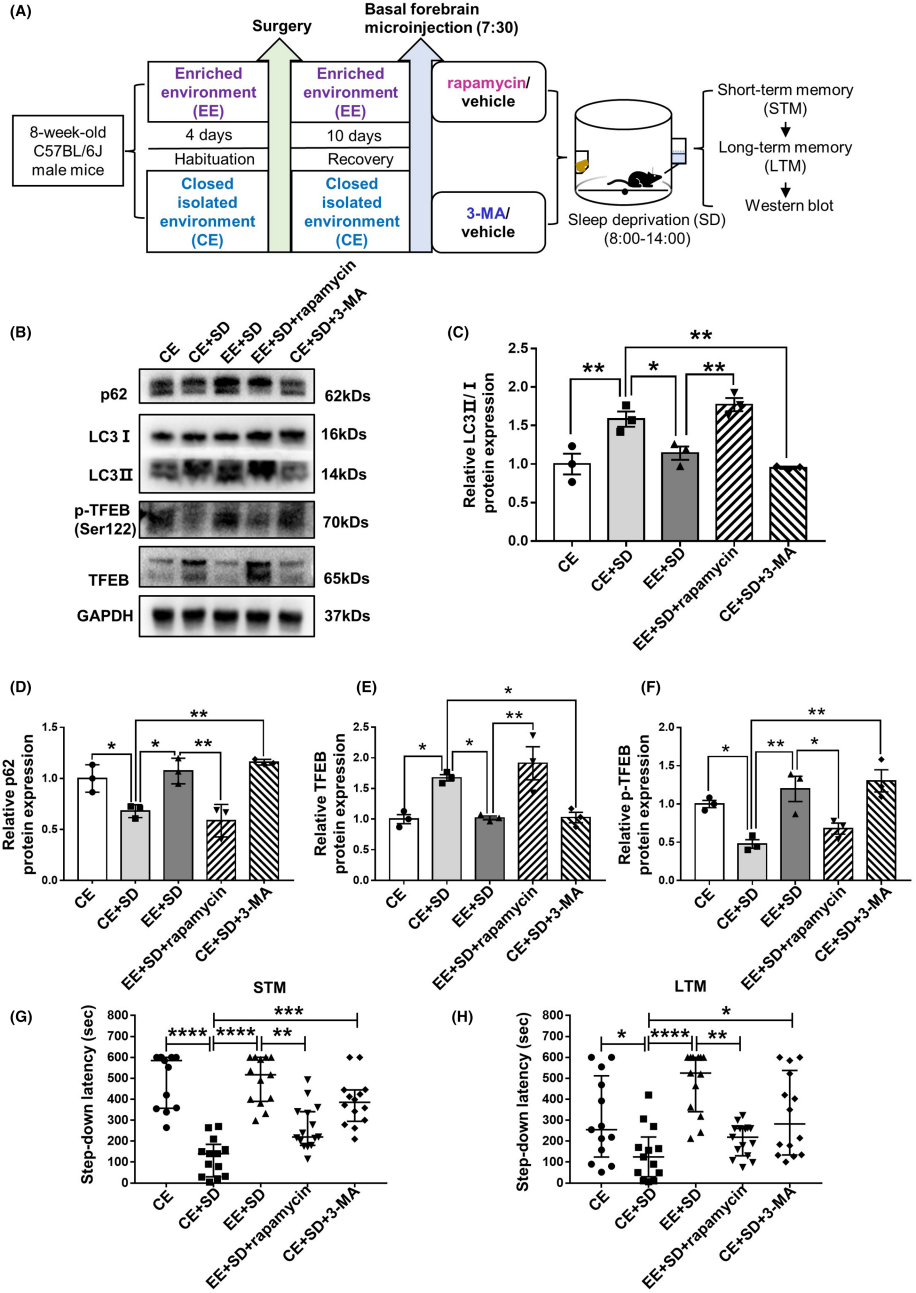
EE inhibits excessive autophagy in the basal forebrain due to sleep deprivation to protect fear memory. (A) Experimental design. (B–F) Changes in autophagy‐related protein expression across groups. Rapamycin abolishes the inhibitory effect of EE on excessive autophagy caused by sleep deprivation, as evidenced by increased LC3 II/I ratio (B, C), decreased p62 protein expression (B, D), increased TFEB protein levels (B, E), and decreased TFEB phosphorylation levels (B, F). 3‐MA mimicked the trend of reduced LC3 II/I ratio (B, C), increased p62 (B, D), reduced TFEB (B, E), and increased TFEB phosphorylation levels (B, F) induced by EE. Changes of short‐term (G) and long‐term (H) fear memory after modulation of autophagic signaling. Thirteen mice each in the CE, CE + SD, and EE + SD groups. Fifteen mice in the EE + SD + rapamycin group, and 14 mice in the CE + SD + 3‐MA group were used. Western blot experiments were performed in three independent samples. LC3 II and LC3 I blots were developed separately. Values are expressed as medians (interquartile ranges) or means ± SEMs. *p < 0.05; **p < 0.01; ****p < 0.0001.

This study was conducted in four stages. First, the effects of the EE on sleep deprivation‐induced memory impairment and changes in basal forebrain PIEZO1 signaling activity were examined. According to their grouping, the mice were housed in either a CE or an EE. Six‐hour total sleep deprivation was imposed on both CE and EE mice. The short‐ and long‐term fear memories were then examined. After fear memory evaluation, the mice were sacrificed, and basal forebrain PIEZO1 signaling changes were detected (Figure 1A). Second, under the premise that EE inhibits the enhanced PIEZO1 protein expression caused by sleep deprivation, the PIEZO1 activator Yoda1 was microinjected into the bilateral basal forebrain of EE mice to observe whether the activation of PIEZO1 signaling could eliminate the cognitive protection of EE under sleep deprivation (Figure 2A). Third, to observe the role of the inhibition of the PIEZO1/calpain signaling pathway in EE‐induced cognitive protection against sleep deprivation, GsMTx4, an inhibitor of PIEZO1 signaling, PD151746, an inhibitor of calpain, or vehicle control was microinjected into the basal forebrain of CE mice. Fear memory performance and basal forebrain PIEZO1 signaling changes were evaluated (Figure 3A). Finally, autophagy in the basal forebrain was examined to determine whether EE ameliorated sleep deprivation‐induced cognitive impairments by inhibiting excessive autophagy (Figures 4 and 5).

### 2.3 Closed isolated environment

The mice were housed individually in plastic mouse cages (32 × 21 × 18 cm) with no other mice within 100 cm of the cage and without any physical or sensational stimulation.^28^

### 2.4 Enriched environment

Various pet toys, including small houses, seesaws, rainbow channels, swings, L‐shaped tunnels, crawling tubes, inclined ladders, fitness rings, and running wheels, were placed in clear acrylic cages (40 × 30 × 40 cm). They were changed each morning with new toys of different colors and shapes to stimulate learning and active exploration.^29^ Five mice were housed per cage to ensure proper socialization. The mice were placed in enriched environment cages for 2 weeks to learn and adapt before subsequent experiments.

### 2.5 Acute sleep deprivation

Commencing with entry into the daytime cycle, mice were subjected to 6 h of complete sleep deprivation using a sleep deprivation apparatus in biofeedback mode (XR108S, Shanghai XinRuan Information Technology Co., Ltd.).^20^ The sleep deprivation efficiency of this apparatus has been previously validated.^30^ During this process, mice moved naturally and had free access to food and water. The apparatus monitored the animal behaviors and activities, and when mice closed their eyes or stopped moving, the percussion bar started to rotate to disturb them and force them to stay awake. EE mice maintained novelty by decorating the apparatus with trinkets during sleep deprivation.

### 2.6 Step‐down inhibitory avoidance test

The apparatus was a 50 × 25 × 25 cm plastic cube box. The bottom of the normal platform consisted of an electrically energized copper grid, and the bottom center had a fixed jumping platform, which was a rubber cylinder 8 cm in diameter and 3 cm above the normal platform. When sleep deprivation was complete, the mice were placed at the center of the platform. When the instinct of exploration drove the mice to climb down the platform, and all their limbs touched the copper grids, they were subjected to an electric shock (0.4 mA lasting 2 s), which caused them to jump back to the platform. The mice's short‐ and long‐term fear memory was measured 1 h and 24 h after training, respectively. During the test phase, the copper grid was not energized.^20^ The time spent on the jumping platform (latency) after the electric shock was used as an indicator of fear memory retention. A longer latency indicates better memory. The upper limit of the latency was set at 600 s.

### 2.7 Stereotactic surgery

The mice were fixed on a stereotaxic instrument (Yu Yan, Shanghai, China) under general anesthesia with sevoflurane. Local infiltration of ropivacaine (0.5%, 1 mL, AstraZeneca) was performed before the incision. After dura exposure, guide cannulas (Cat#62524, RWD) with a length of 4.5 mm were inserted 1 mm above the bilateral basal forebrain (anterior–posterior (AP)—0.01 mm, medial‐lateral (ML) ±1.5 mm, and profundity dorsal‐ventral (DV) – 5.5 mm). The double‐lumen catheters have an O.D. of 0.48 mm and I.D. of 0.34 mm for each lumen (Cat#62036, RWD). The interluminal distance was 2 mm. The catheter was secured to the skull using bone cement.

### 2.8 Microinjection of drugs

Under sevoflurane anesthesia, the catheter cap was unscrewed from the mouse and the pin was removed. A polyethylene tube (Cat#62329, RWD) at a length of about 1.5 cm connects the inner injection tubing (O.D. 0.3 mm × I.D. 0.14 mm) to the 2 μL microinjector (Cat#79003, RWD) containing the drug. The drug was slowly injected into the bilateral basal forebrain within 2 min, and after the injection was completed, the inner injection tube was left in place for 3 min to allow the drug to diffuse. The drugs used were Yoda1 (250 ng/250 nL/side; Cat#HY‐18723, MCE), GsMTx4 (300 ng/250 nL/side; Cat#P1205, Selleck.cn), PD151746 (2 μg/250 nL/side; Cat#HY‐19749, MCE), rapamycin (0.7 ng/250 nL/side; Cat#146990, TargetMol), 3‐MA (1357 ng/250 nL/side, Cat#5142‐23‐4, Sigma‐Aldrich) and their vehicle control. The accuracy of the injection sites was assessed by visual inspection or DAPI staining. Only data from the mice with the correct injection sites were used.

### 2.9 Tissue extraction for Western blot and PCR

The whole brain tissue was removed and the optic cross was identified. One millimeter‐thick coronal sections were taken in front of and behind the optic cross. Horizontal sections were obtained at the optic cross and anterior union, and the ventral brain tissue was preserved. Sagittal sections were made at 1 mm lateral to the third ventricle and at the midpoint of the olfactory bulb. Tissue (1 mm × 1 mm × 2 mm) was frozen at −80°C for subsequent experiments.^20^

### 2.10 Western blot

The collected basal forebrain tissues were placed in a grinding tube. Tissue lysate and protease phosphatase inhibitor (100:1 volume ratio) were added for lysis at 4°C for 30 min. After centrifugation at 4°C (16,320 *g*) for 20 min, the supernatant was retained and protein concentration was measured using BCA Protein Assay Kit (Cat# P0012, Beyotime). Equal amounts of protein were subjected to electrophoresis (185 V for 50 min for all experiments). Separated proteins were transferred onto polyvinylidene fluoride membranes (Cat#10600023, GE). After blocking, the membrane was incubated with primary antibody overnight at 4°C in a shaker. The membrane was washed three times with 1 × TBST solution, followed by incubation with an HRP‐conjugated secondary antibody (Cat#SA00001‐1; Cat#SA00001‐2, Proteintech) for 1 h at room temperature. Blots were developed using a commercial ECL kit. Luminescence was captured using a gel imaging system (Bio‐Rad ChemiDoc™ MP). The optical density of the bands was determined using ImageJ software, with GAPDH or Tubulin as internal controls.^20^ The antibodies used were as follows: rabbit anti‐PIEZO1 antibody (1:1000, Cat#A4340, Abclonal), rabbit anti‐phosphorylated TrkB (Tyr516) (p‐TrkB) (1:1000, Cat#4619S, Cell Signaling Technology (CST)), rabbit anti‐TrkB (1:1000, Cat#ab18987, Abcam), rabbit anti‐phosphorylated PLCγ1 (p‐PLCγ1) (Tyr783) (1:1000, ab134186, Abcam), rabbit anti‐PSD95 (1:1000, Cat#ab18258, Abcam), rabbit anti‐LC3B (1:1000) (Cat#ab192890, Abcam), rabbit anti‐p62 (1:1000, Cat#23214S, CST), rabbit anti‐TFEB (1:1000) (Cat# 13372‐1‐AP, Proteintech), rabbit anti‐phosphorylated TFEB (Ser122) (p‐TFEB, 1:1000, Cat#86843S, CST), rabbit anti‐GAPDH (1:1000, Cat# 60004‐1‐Ig, Proteintech), and rabbit anti‐Tubulin (1:1000, Cat#66031‐1‐Ig, Proteintech).

### 2.11 Tissue fixation and immunofluorescence

After the long‐term fear memory test, the mice were first perfused with cold saline and then with 4% paraformaldehyde for fixation. After fixation, the whole brain was taken and submerged in 4% paraformaldehyde at 4°C for long‐term storage. For immunofluorescence, the slices were sectioned using a frozen sectioning machine (Leica) with a thickness of 20 μm. The sectioned tissues were blocked with 10% goat serum at 37°C for 1 h and then incubated with rabbit anti‐LC3B antibody (1:200, Cat#ab192890, Abcam) or anti‐rabbit TFEB antibody (1:200, Cat#13372‐1‐AP, Proteintech) overnight at 4°C. After washing, Cy3 conjugated Donkey Anti‐Rabbit IgG (H + L) (1:50, Cat# GB21403, Servicebio) or FITC‐conjugated Goat Anti‐Rabbit IgG (H + L) (1:50, Cat# GB22303, Servicebio) was added. After incubation for 1 h, DAPI (1:500, Cat# P0131, Beyotime) was added to the slices for 5 min to visualize the nuclei. The images were scanned using a Pannoramic Desk Scanner (3DHistech), viewed, and cropped using CaseViewer software (version 2.3).

### 2.12 Real‐time quantitative PCR

Total RNA was extracted from the mouse basal forebrain according to the TRIzol Reagent instructions. mRNA was reverse transcribed to cDNA using PrimeScript™ RT reagent kit with gDNA Eraser (Takara) as a template, and then amplified on a PCR instrument (Bio‐Rad), and finally the raw results were obtained from Bio‐Rad CFX Manager software and then analyzed using the ${{2^{-}}^{\Delta \Delta }}^{C_{t}}$ method. The primer sequences used were as follows: PIEZO1, Forward: 5′‐CGGACAGTGAGGAGGAAGAGGAG‐3′ and Reverse: 5′‐CCTGTTCACGACGACGCTGCCTTAG‐3′; p62, Forward: 5′‐GGATGGGGACTTGGTTGC‐3′ and Reverse: 5′‐TCACAGATCACATTGGGGTGC‐3′; GAPDH, Forward: 5′‐CAGTGCCAGCCTCGTCTCAT‐3′ and Reverse: 5′‐AGGGGCCATCCACAGTCTTC‐3′.^31^

### 2.13 Statistical analyses

GraphPad Prism 7 and SPSS 26.0 software (IBM) were used for data analysis and graphing. The Shapiro–Wilk test was used for assessing measurement data normality. For normality data (PCR and Western blot data in this study), they were expressed as means ± standard errors (SEMs) and were compared using independent samples *t*‐test or one‐way ANOVA followed by post hoc Tukey pairwise comparison. For data not conforming to normality (fear memory data), they were expressed as medians (interquartile ranges), and Kruskal‐Wallis non‐parametric test with post hoc Bonferroni correction for multiple comparisons was used for comparison. For all the analyses, *p* < 0.05 (two sides) indicates a statistically significant difference.

## 3 RESULTS

### 3.1 Enriched environment ameliorates fear memory impairments by sleep deprivation and reverses enhanced PIEZO1 expression

The experimental design is shown in Figure 1A. In the step‐down fear memory test, significant statistical differences were found among the three groups for both short‐ (*p* < 0.0001) and long‐term (*p* = 0.0042) memory performance. Specifically, acute sleep deprivation resulted in impaired short‐ (Figure 1B; *p* = 0.0020) and long‐term (Figure 1C; *p* = 0.038) memory in mice housed in a CE compared to the control. Both short‐ (*p* < 0.0001) and long‐term (*p* = 0.0055) memory capacities were superior in mice reared in an EE compared to those in CE mice. Sleep deprivation significantly increased PIEZO1 mRNA transcription (Figure 1D; *p* < 0.0001 vs. control), which was partially reversed by EE treatment (*p* = 0.0060 vs. CE plus sleep deprivation). EE also suppressed the sleep deprivation‐induced increase in PIEZO1 protein expression (Figure 1E; *F*_(2, 6)_ = 6.4920, *p* = 0.0467 vs. CE plus sleep deprivation). Compared with control, sleep deprivation did not affect TrkB‐FL protein expression (Figure 1F; *p* = 0.1951), but significantly increased the levels of TrkB‐ICD, a marker of TrkB‐FL degradation (Figure 1G; *p* = 0.0084) and decreased PLCγ1 phosphorylation, a downstream marker of BDNF/TrkB‐FL signaling (Figure 1H; *p* = 0.0450). The EE partially alleviated these effects (all *p* < 0.05).

### 3.2 PIEZO1 activator eliminates the ameliorative effect of EE on fear memory impairments due to sleep deprivation

The PIEZO1 activator Yoda1 (250 ng/250 nL) or vehicle (Figure 2A–C) was injected into the bilateral basal forebrain of the mice before sleep deprivation. In EE mice, Yoda1 injection caused a significant decrease in short‐term (Figure 2D; *p* < 0.0001) and long‐term (Figure 2E; *p* < 0.0001) memory. PIEZO1 protein levels were significantly increased following Yoda1 treatment (Figure 2F and Figure S1A; *p* = 0.0338). TrkB‐FL quantity was not changed (Figure 2F and Figure S1B; *p* = 0.7310), but TrkB‐ICD formation was increased (Figure 2F and Figure S1C; *p* = 0.0408), and downstream PLCγ1 activation was decreased (Figure 2G and Figure S1D; *p* = 0.0026).

### 3.3 Inhibition of PIEZO1/calpain signaling mimics the ameliorative effect of EE on sleep deprivation‐induced fear memory impairments

The effects of inhibiting PIEZO1/calpain signaling on sleep deprivation‐induced fear memory impairments were further investigated (Figure 3A). Subgroup analysis found that the memory performances of mice injected with the PIEZO1 inhibitor GsMTx4 and the calpain inhibitor PD151746 were better than those of mice injected with the vehicle. Short‐ (Figure 3B; *p* < 0.0010; *p* = 0.0016) and long‐term (Figure 3C; *p* < 0.0001; *p* = 0.0015) fear memories were all improved by GsMTx4 or PD151746. Western blot results showed decreased PIEZO1 protein expression (Figure 3D,E; *p* = 0.0032; *p* = 0.7568), increased TrkB phosphorylation (Figure 3D,F; *p* = 0.0317; *p* = 0.0405), and decreased TrkB‐ICD formation (Figure 3G,H; *p* = 0.0116; *p* = 0.0253) in response to GsMTx4 and PD151746 (all vs. CE plus sleep deprivation). Downstreaming PLCγ1 activation (Figure 3G,I; *p* = 0.0293; *p* = 0.0450) and the protein expression of synaptic plasticity‐related gene PSD95 (Figure 3G,J; *p* = 0.0084; *p* = 0.0213) were increased by GsMTx4 and PD151746 (all vs. CE plus sleep deprivation).

### 3.4 EE inhibits excessive autophagy in the basal forebrain due to sleep deprivation to protect fear memory

Western blot results showed (Figure 4A) that the basal forebrain LC3 II/I protein ratio increased (Figure 4A and Figure S2A; *p* = 0.0326), p62 protein decreased (Figure 4A and Figure S2B; *p* = 0.0150), TFEB expression increased (Figure 4B and Figure S2C; *p* = 0.0115), and TFEB phosphorylation decreased (Figure 4B and Figure S2D; *p* = 0.0489) after sleep deprivation compared with the control. p62 is a target gene of TFEB. The PCR results showed that p62 mRNA expression increased in CE mice after sleep deprivation, which was reversed by EE (Figure 4C). LC3B (Figure 4D) and TFEB (Figure 4E) immunofluorescence staining showed similar results to the Western blot findings and increased TFEB nuclear localization, indicating excessive autophagy. EE treatment partially reversed these changes (all *p* < 0.05). Basal forebrain injection of the PIEZO1 activator Yoda1 increased autophagy in EE mice, with an elevated LC3 II/I ratio (Figure 4F and Figure S2E; *p* = 0.0354), decreased p62 protein levels (Figure 4F and Figure S2F; *p* = 0.0013), increased TFEB protein levels (Figure 4F and Figure S2G; *p* = 0.0081), and decreased TFEB phosphorylation (Figure 4F and Figure S2H; *p* = 0.0361). Microinjections of either GsMTx4 or PD151746 resulted in decreased LC3 II/I ratio (Figure 4G and Figure S2I; *p* = 0.0031; *p* = 0.0119), increased p62 protein expression (Figure 4G and Figure S2J; *p* = 0.0005; *p* = 0.0355), decreased TFEB protein levels (Figure 4G and Figure S2K; *p* = 0.0310; *p* = 0.0352), and increased TFEB phosphorylation (Figure 4G and Figure S2L; *p* = 0.0020; *p* = 0.0129) compared with vehicle control in sleep‐deprived mice. To demonstrate that EE improves fear memory improvements by inhibiting excessive autophagy due to sleep deprivation, the autophagy inducer rapamycin and inhibitor 3‐MA were injected bilaterally into the basal forebrain (Figure 5A). For EE mice, rapamycin‐induced strong autophagy evidenced by increased LC3 II/I ratio (Figure 5B,C; *p* = 0.0049), enhanced p62 degradation (Figure 5B,D; *p* = 0.0026), increased TFEB expression (Figure 5B,E; *p* = 0.0058), decreased TFEB phosphorylation (Figure 5B,F; *p* = 0.0442) and significant defects in both short‐(Figure 5G; *p* = 0.0069 and long‐(Figure 5H; *p* = 0.0056) term memory. In CE mice, 3‐MA reduced the LC3 II/I ratio (Figure 5B,C; *p* = 0.0048), inhibited p62 degradation (Figure 5B,D; *p* = 0.0029), decreased TFEB expression (Figure 5B,E; *p* = 0.0405), enhanced TFEB phosphorylation (Figure 5B,F; *p* = 0.0023) and improved both short‐(Figure 5G; *p* = 0.0006) and long‐term memory (Figure 5H; *p* = 0.0482).

## 4 DISCUSSION

Neural networks in the brain are unstable and highly susceptible to sleep deprivation interference, resulting in decreased attention and memory.^32^ The basal forebrain not only regulates sleep–wake transitions but also participates in regulating cognitive functions.^33^ George et al. found that sleep duration decreased with increasing age in rats and that decreased basal forebrain progesterone levels were associated with age‐related sleep‐dependent memory impairments.^34^ In addition, patients with Alzheimer's disease have significant sleep fragmentation and sleep deprivation, and sleep disturbances occur before cognitive impairments,^5^ but have a similar onset time to basal forebrain degeneration.^35^, ^36^ These findings suggest that sleep deprivation leads to cognitive impairment in the basal forebrain. Therefore, clarifying the basal forebrain regulatory mechanism of sleep deprivation‐induced cognitive impairment may help combat sleep deprivation‐induced cognitive impairment and provide new directions for the prevention and treatment of Alzheimer's disease.

Alterations in Ca^2+^ homeostasis are also associated with sleep deficiency and cognitive impairment. Astroglial Ca^2+^ signals are highest in the wake and lowest in sleep, and synchrony decreases during sleep compared to that during the wake and after sleep deprivation.^37^ Sleep deprivation reduces calcium intensity and NMDAR1/nNOS expression in the rat hippocampus, which is accompanied by poor performance in behavioral memory testing.^38^ Moreover, disrupted calcium buffering in the basal forebrain cholinergic neurons may be associated with cognitive decline in older patients.^39^, ^40^ PIEZO1 acts mainly through Ca^2+^/calpain signaling.^41^ Calpain, a Ca^2+^‐dependent non‐lysosomal cysteine protease,^42^ is a key molecule in the cognitive impairment caused by sleep deprivation. Our previous study showed that basal forebrain PIEZO1 expression increases after sleep deprivation.^20^ Furthermore, inhibition of PIEZO1/calpain signaling in the basal forebrain partly reversed sleep deprivation‐induced fear memory deficits.^20^ The current study further found that the inhibition of PIEZO1/calpain signaling contributed to the cognitive protective effects of the EE in the context of sleep deprivation (Figures 2 and 3). This evidence verifies the role of increased PIEZO1 signaling in sleep deprivation‐induced memory impairment. However, we did not have the related equipment and technology to measure the mechanodynamic changes in basal forebrain neurons after sleep deprivation, and why sleep deprivation increased the expression of PIEZO1 was not explored. However, further experiments are required to address this issue.

An EE means more room to move, more objects to explore, entertain, and exercise, and greater motor and sensory stimulation than a CE, which may help maintain cognitive stability or reduce cognitive impairment due to brain injury.^43^, ^44^ Similar to a previous report,^29^ the present study found that EE improved cognitive impairments following acute sleep deprivation (Figure 1). Further experiments found that activation of basal forebrain PIEZO1 abrogated the cognitive protective effect of EE (Figure 2), suggesting that the basal forebrain and PIEZO1 signaling may be the possible brain regions and signaling mechanisms of EE‐induced memory maintenance. The results of several previous studies support this finding. For example, EE increases the number of cholinergic‐positive neurons in the medial compartment of the basal forebrain to counteract spatial learning and memory impairment after traumatic brain injury.^16^ In post‐stroke mice, EE increased basal forebrain and hippocampal ChAT protein expression, increased acetylcholine levels, and improved learning and memory.^17^ Combined with previous reports that sleep deprivation impairs calcium signaling^37^, ^38^ and that EE inhibits hyperactivation of calcium signaling,^45^ we speculated that EE might counteract acute sleep deprivation‐induced cognitive impairment by modulating basal forebrain/PIEZO1/calcium signaling.

Both acute sleep deprivation^19^ and EE increased BDNF protein expression,^43^ promoting basal forebrain acetylcholine release and regulating learning and memory.^46^ BDNF binds to the TrkB receptor for signal transduction. We have previously found that acute sleep deprivation in mice induces fear memory impairment by increasing TrkB degradation due to calpain activation, which decreases the efficiency of BDNF signaling.^20^ In the present study, we found that both PIEZO1 and calpain inhibition decreased TrkB degradation and maintained memory performance similar to EE (Figure 3), suggesting that EE could counteract the cognitive impairment caused by sleep deprivation by inhibiting PIEZO1/calpain signaling. However, calpain activity in the basal forebrain has not been examined directly, and only changes in TrkB cleavage have been measured. This study did not further investigate whether the activation of basal forebrain calpain, which promotes TrkB degradation, could lead to cognitive impairments similar to those of sleep deprivation.

Both excessive and reduced autophagy is associated with cognitive impairments.^47^ Acute sleep deprivation causes autophagy in hippocampal neurons. Similarly, we found that acute sleep deprivation promoted basal forebrain autophagy, which was reversed by EE (Figure 4). Activation of PIEZO1 eliminated the cognitive protective (Figure 2) and autophagy‐inhibitory effects of EE (Figure 4). Conversely, the inhibition of PIEZO1 or calpain ameliorated cognitive impairment and autophagy was similarly inhibited. This suggests that autophagy is involved in acute sleep deprivation‐induced fear memory impairment. We further found that the autophagy inducer rapamycin eliminated the protective effects of EE on cognition. Conversely, inhibiting autophagy using 3‐MA ameliorated fear memory impairment caused by acute sleep deprivation, mimicking the protective cognitive effects of EE (Figure 5). These results further confirmed that EE ameliorated fear memory impairment by suppressing excessive autophagy caused by sleep deprivation. However, we did not observe whether autophagy occurred in neurons or specific types of neurons.

TFEB is a major regulator of the autophagy‐lysosomal pathway.^48^ A localized increase in intracellular Ca^2+^ induces TFEB dephosphorylation and promotes nuclear ectopic and autophagic activation of TFEB.^26^ Consistent with these findings, we found that PIEZO1 activation by Yoda 1 and sleep deprivation was associated with increased TFEB expression, decreased TFEB phosphorylation, and increased TFEB nuclear translocation (Figure 4). The PIEZO1 inhibitor GsMTx4 partly reversed the change in TFEB signaling and excessive downstream autophagy. We further found that the calpain inhibitor PD151746 inhibited TFEB expression and dephosphorylation (Figure 4G). A previous study found that a lack of Pkd1 led to the impairment of autophagosomal‐lysosomal fusion through increased activity of calpain without affecting the TFEB‐dependent lysosomal biogenesis pathway.^49^ In contrast, PIEZO1 activated calpain/calcineurin signaling to promote cardiac hypertrophy,^50^ and calcineurin could bind and dephosphorylate TFEB, thereby promoting its nuclear translocation.^26^ It would be interesting to further clarify whether there is a regulatory role for PIEZO1/calpain in TFEB signaling. One limitation is that we did not observe whether inhibition of TFEB expression or inhibition of TFEB nuclear translocation could counteract the cognitive impairment caused by sleep deprivation, and the expression changes of autophagy‐related genes by TFEB, except for p62, were not examined.

In summary, an enriched environment helps counteract sleep deprivation‐induced fear memory impairments in mice by inhibiting the basal forebrain PIEZO1/calpain/autophagy pathway. Modulation of basal forebrain PIEZO1 signaling by pharmacological or non‐pharmacological means may be an effective strategy to counteract sleep deprivation‐induced cognitive impairment.

## CONFLICT OF INTEREST STATEMENT

The authors declare no existing or potential conflict of interest.

## Supporting information


Figures S1

## Data Availability

The data supporting the results are available from the corresponding author upon reasonable request.
