# Supplementary material for: Enriched environment ameliorates fear memory impairments induced by sleep deprivation via inhibiting PIEZO1/calpain/autophagy signaling pathway in the basal forebrain
Source: CNS Neurosci Ther. 2023 Jul 23;30(2):e14365. doi: 10.1111/cns.14365 (PMC10848088; doi:10.1111/cns.14365)
Supplement: Supplementary file 1 — Figures S1 [file CNS-30-e14365-s001.docx]

**Supplementary materials**

**Figure S1**


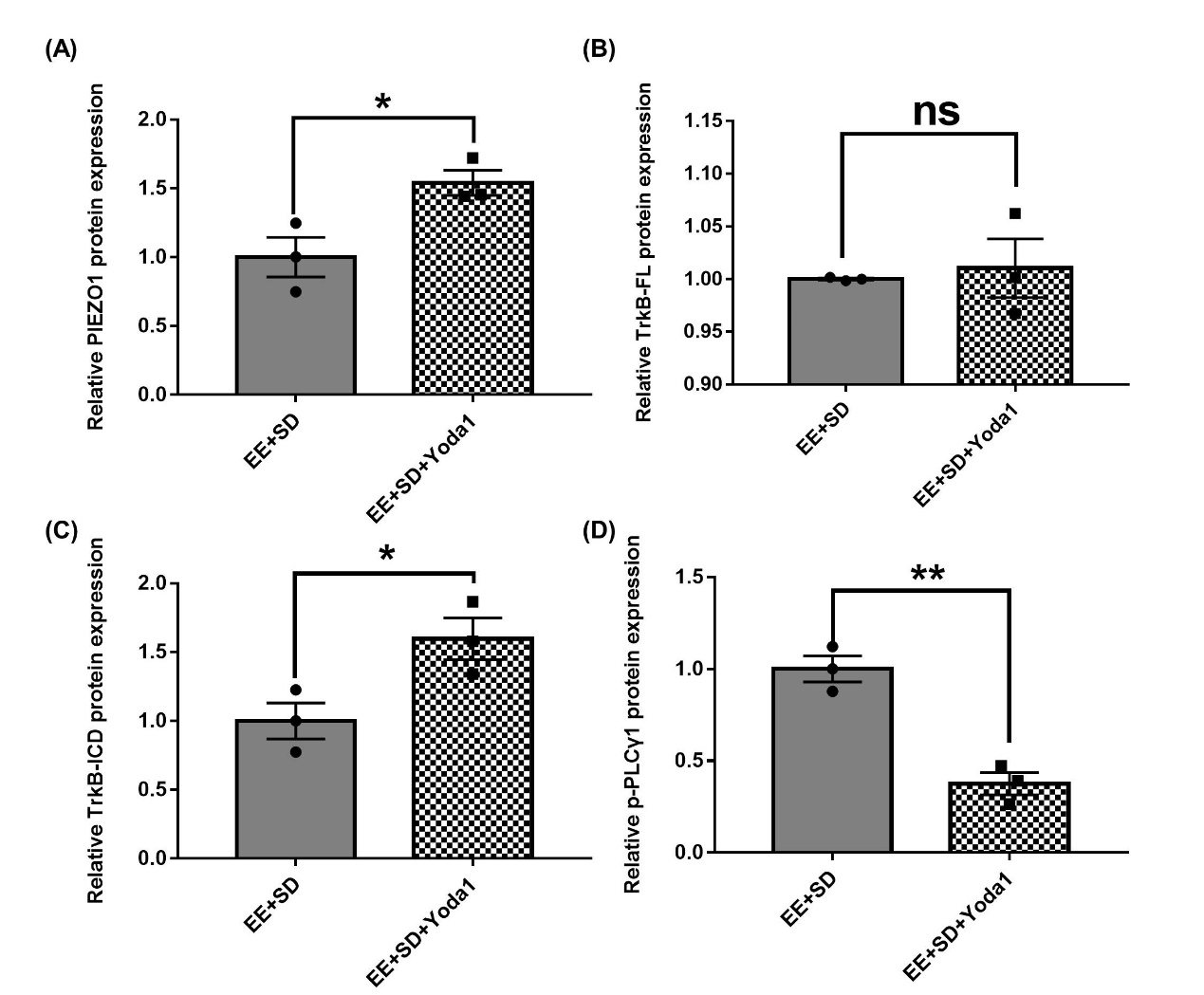


**Figure S1. Changes in expression of PIEZO1 (A), full length TrkB (TrkB-FL, B), TrkB cleavage prodcut by calpain (TrkB-ICD, C) and phosphorylated PLCγ1 (D) after the PIEZO1 activator Yoda1 injection in mice raised in enriched environment (EE) and receiving sleep deprivation (SD).** *, p < 0.05; and **, p < 0.01. ns: no significant difference.

**Figure S2**


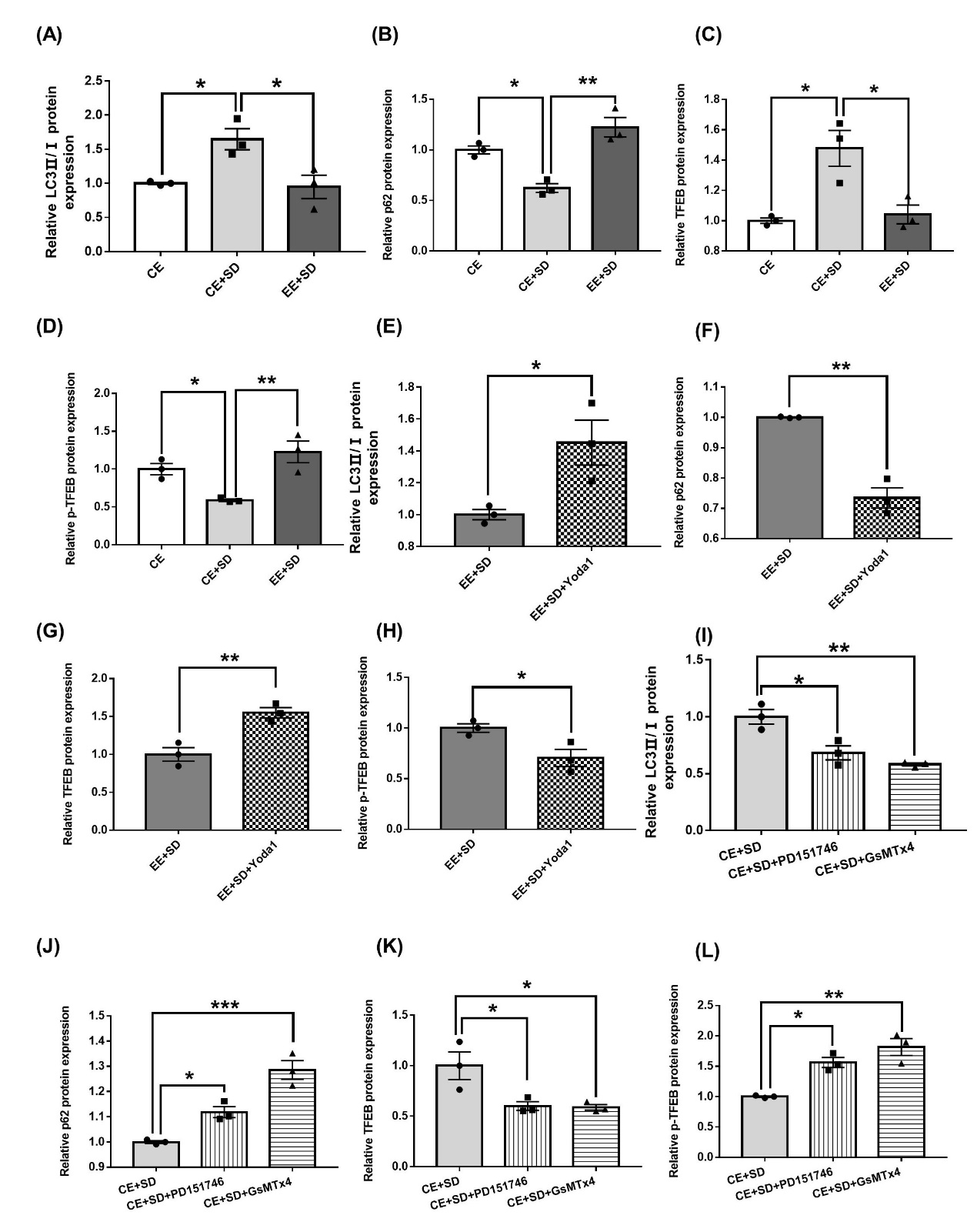


**Figure S2.** **Enriched environment (EE) or inhibition of PIEZO1/calpain signaling alleviates excessive autophagy caused by sleep deprivation (SD).** (**A to D)** Histograms of autophagy marker protein expression in mice raised closed isolated environment (CE) with/without SD and mice raised in EE with SD. (**E to H)** Histograms of autophagy marker protein expression in sleep-deprived EE mice receiving the PIEZO1 activator Yoda1 injection. **(I to L**) Histograms of autophagy marker protein expression in sleep-deprived CE mice receiving the PIEZO1 inhibitor GsMTx4 or the calpain inhibitor PD151746 microinjection. *, p < 0.05; **, p < 0.01 and ***, p < 0.001.
